# Screening tuberculosis patients for diabetes mellitus in a routine program setting in Kampala, Uganda: a cross-sectional study

**DOI:** 10.12688/f1000research.19279.2

**Published:** 2019-10-10

**Authors:** Joseph Nsonga, John Paul Dongo, Frank Mugabe, Gerald Mutungi, Richard Walyomo, Christopher Oundo, Sarah Zalwango, Daniel Okello, Simon Muchuro, Riitta A Dlodlo, Yan Lin

**Affiliations:** 1 International Union Against Tuberculosis and Lung Disease, Plot 2, Lourdel Road, Nakasero Hill, Kampala, Uganda; 2 The National Tuberculosis and Leprosy Program, Ministry of Health, Plot 6, Lourdel Road, Nakasero, Kampala, Uganda; 3 The Non-Communicable Diseases Program, Ministry of Health, Plot 6, Lourdel Road, Nakasero, Kampala, Uganda; 4 Kampala Capital City Authority, City Hall, Plot 1-3, Apollo Kaggwa Road, Kampala, Uganda; 5 University Research Company on USAID Defeat Project, Plot 40 Ntinda II Road, Naguru, Kampala, Uganda; 6 International Union Against Tuberculosis and Lung Disease, 68 Boulevard Saint-Michel, Paris, 75006, France

**Keywords:** Tuberculosis, Diabetes Mellitus, Screening, Uganda

## Abstract

**Background**: Uganda is located in East Africa and is among the countries with the lowest income globally. The ten health centres in this project serve populations in the under-privileged communities of Kampala. The objective of the study was to implement diabetes mellitus (DM) screening among tuberculosis (TB) patients in a routine program setting with limited resources and high human immunodeficiency virus (HIV) prevalence.

**Methods**: A descriptive cross-sectional observational study was conducted in ten health centres in Kampala, Uganda. As part of a project to implement DM screening in a routine setting, TB patients were screened for DM by trained health workers. A fasting blood glucose (FBG) value ≥7.0mmol/l was considered to indicate DM. For this study, aggregate data was collected and analysed using SPSS for Windows, version 13.0.

**Results**:  Among 4,590 TB patients registered, 4,016 (88.0%) were screened with random blood glucose (RBG). Of those with RBG ≥6.1mmol/l, 1,093 (83.3%) were screened with FBG. In total, 92 (2.3%) patients were diagnosed with DM and 66 (71.8%) of them were newly diagnosed. The proportion of TB patients screened with FBG in the health centres varied from 58.2% to 100%. The proportion of patients screened with FBG and the prevalence of DM were significantly higher in private health centres compared with public health centres. The health centres in peri-urban areas screened more patients with RBG than those in urban areas. These health centres without DM services screened a larger number of patients with RBG and FBG than those with DM services.

**Conclusions**: It appears feasible to implement screening TB patients for DM in routine program settings with limited resources and high HIV prevalence. Its introduction requires close collaboration between TB and DM services. The challenges identified need government attention and certain institutional and service-related factors need to be better managed at times

## Introduction

Although significant progress has been achieved in tuberculosis (TB) care and prevention during the past decades, TB remains a major public health problem and is responsible for more deaths than any other single infectious disease worldwide ^1^. In 2017 globally, 10.0 million people developed TB and 1.6 million died from it ^1^. Uganda is among the 30 high TB and human immunodeficiency virus (HIV) burden countries, with an estimated TB prevalence of 253 per 100,000 population (95% CI: 196-317) and 40% HIV co-infection rate ^2, 3^.

Along with socio-economic development, urbanization, dietary and lifestyle changes, the prevalence of diabetes mellitus (DM) is escalating in most low- and middle-income countries. Available data suggests that an estimated 425 million people worldwide live with DM and by 2045 this number will grow to 629 million ^4^. Although estimates for sub-Saharan Africa are based on limited data with substantial uncertainty, DM appears to be increasing rapidly in many urban centres in the region ^5^. In Uganda, some risk factors for DM, such as obesity, were greater among persons of a high socio-economic status in rural areas, though the prevalence of DM in the adult population of the entire country was relatively low (1.4% in a nationwide cross-sectional survey in 2014) ^6, 7^.

DM is a well-known risk factor for TB and increases its risk 2-3-fold ^8, 9^. Systematic reviews and meta-analyses suggest that DM patients with TB experience worse treatment outcomes compared with patients without DM, with delayed sputum smear conversion, increased risk of drug resistance, treatment failure, death and relapse after successful completion of anti-TB treatment ^8– 12^. On the other hand, TB patients with previously known DM are at an increased risk of hyperglycemia or worse glycaemic control ^13, 14^. Therefore, early detection of DM among TB patients and proper management of both conditions are well known measures to improve treatment outcomes in these patients.

To address this problem in low- and middle-income countries, the World Health Organization (WHO) and the International Union Against Tuberculosis and Lung Disease (The Union) launched a ‘Collaborative Framework for the Care and Control of Diabetes and Tuberculosis’ in 2011, and recommended bi-directional screening for TB and DM within routine health services ^15^.

The Union, together with national authorities, implemented the first bi-directional screening of DM and TB within routine health services in China and India, where it was found to be feasible in these settings ^16– 18^. In Uganda, there is no policy on routine screening for DM among TB patients. Whether a similar approach can be implemented in a resource limited setting with a high HIV prevalence remained unknown. To further understand this, a project was implemented from 2016 to 2017 to screen TB patients for DM, supported by the World Diabetes Foundation (WDF). Objectives of this study were to: a) assess whether DM screening can be implemented among TB patients in a routine programmatic health setting in Uganda; b) identify challenges for DM screening among TB patients and solutions to overcome them; and c) identify institutional and health service related factors that may have an impact on DM screening among TB patients. 

## Methods

### DM screening project

***Setting and sites. ***Kampala is the capital city of Uganda and had an estimated population of 1,607,182 in 2017. In the same year, the Kampala Capital City Authority (KCCA) notified a total of 7,260 TB cases, resulting in a case notification rate of 452/100,000 population ^3^. Treatment success was 84.6%. HIV prevalence was 6.9% nationwide (Uganda Population-based HIV Impact Assessment UPHIA 2016–2017) ^3^. 

Health services in the city are provided by the KCCA. Persons with presumed TB are investigated in health centres and diagnosed TB patients receive care in TB clinics. DM services are available mainly in specialised DM clinics at tertiary level health facilities.

Ten health centres, both public and private, urban and rural, in Kampala were selected based on their location in the five administrative divisions of KCCA and representative of the health delivery system in Kampala. These were high volume health facilities and selected from different communities based on their ability to manage sufficient numbers of TB patients registered in the previous year and a daily TB patient visits ≥20/day, availability of diagnostic and treatment facilities within the same catchment area and willingness of the staff to participate in this study.

**Figure 1.**
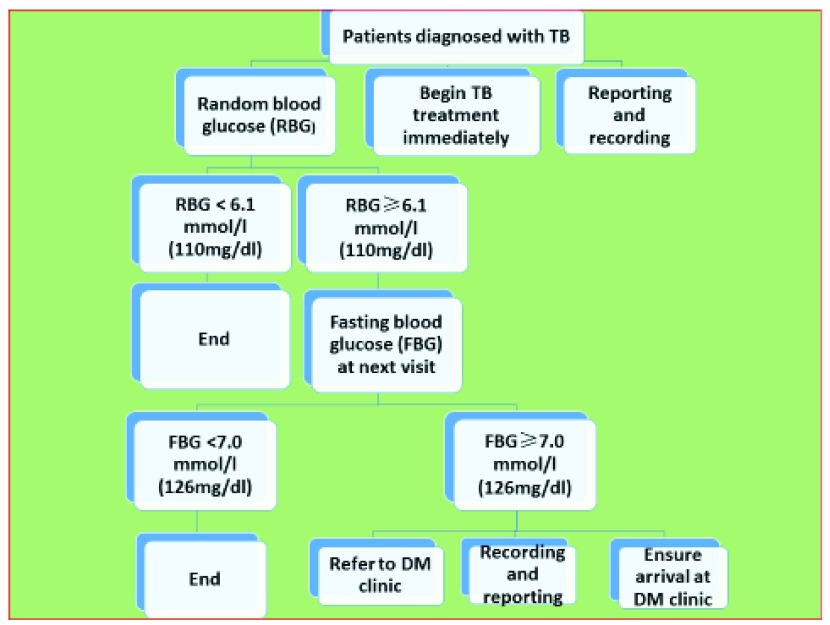
Algorithm for diabetes mellitus (DM) screening among tuberculosis patients in Kampala, Uganda.

***Project training, screening algorithm and monitoring. ***The National TB and Non-Communicable Disease (NCD) Programs’ staff, Kampala Capital City Authority (KCCA) and The Union Uganda Office staff attended an inception meeting and training of trainers in February-March 2016. Participants reviewed the association between DM and TB, the need for screening and agreed to use the screening algorithm shown in Figure 1. Cascade training sessions for 40 staff from the selected health centres were then conducted. The Union and KCCA officials performed quarterly monitoring, compiled periodic reports and provided overall supportive supervision for the activities. At the same time, they recorded any challenge encountered during the implementation.

***Patients. ***Patients aged 15 years and over who were seeking health services in the health centres and were newly registered with any form of TB between 1 ^st^ April 2016 to 31 ^st^ December 2017 were included in the screening project.

***Blood glucose measurement in TB patients. ***A blood sample was collected for random blood glucose (RBG) using capillary blood immediately after the TB registration in the health centres. A OneTouch glucometer (model SN SAFTM7K8, Lifescan, Johnson & Johnson, New Brunswick, USA) was used with a detection value ranging from 1.7 to 19.1mmol/l. Patients with an RBG value ≥6.1mmol/l were invited to return on the following day, after an overnight fast of at least 10 hours, for measurement of fasting blood glucose (FBG). The FBG cut-off thresholds were based on the World Health Organization (WHO) recommendations as follows: i) FBG ≥7.0 mmol/l (≥126 mg/dl) indicates presence of DM; ii) FBG of 6.1 – 6.9 mmol/l (from 110 mg/dl to less than 126 mg/dl) indicates impaired glucose tolerance; and iii) FBG <6.1 mmol/l (<110 mg /dl) indicates normal blood glucose levels. Health workers provided information on the importance of fasting before returning for a confirmatory test (FBG) to the patients through health education. Reminder phone calls were also provided to all patients who screened positive for RBG to return for confirmatory test.

### Study design

This was a descriptive cross-sectional observational study using routine project data from ten health centres in Kampala, Uganda.

### Data collection and analysis

District TB and Leprosy Supervisors (DTLs) collected routine programmatic data every six months from the unit TB and DM registers in the 10 implementing health facilities. Final data collection was finished in December 2017. The Union staff reviewed the data and worked together with the health centre staff to trace the missing data (mainly the number of patients with known DM). The DTLs collected aggregated data, which was verified by The Union staff, and double-entered the data into a spreadsheet. Comparisons of characteristics between patients attending different health centres were carried out using the chi square test with odds ratios (OR) and their 95% confidence intervals. Levels of significance were set at 5%. Statistical analysis was carried out using SPSS for Windows version 13.0 (SPSS Inc., Chicago, IL, USA).

### Ethical statement

This project and the research proposal were approved by the National Tuberculosis and Leprosy Program, the Non-Communicable Diseases Program, Ministry of Health of the Government of Uganda, and Kampala Capital City Authority. As the information collected formed part of routine programmatic health service delivery and no individual patient data was collected, review by an ethics committee and patient consent were not considered necessary.

## Results

The characteristics of the selected health centres are described in Table 1. Of the 4,590 TB patients notified, 4,016 (88.0%) were screened with RBG ^19^. Of those with RBG ≥6.1mmol/l, 1,093 (83.3%) were screened with FBG. Of the 92 TB patients diagnosed with DM, 66 (71.7%) were newly diagnosed DM and were referred to DM services. All DM patients arrived at the DM clinics and received DM care except four patients who died shortly after TB registration. The prevalence of DM in the screened TB patients was 2.3% ( Table 2).

**Table 1.** Characteristics of the 10 study health centres, Kampala, Uganda, from 2016–2017.

Characteristics	Kisenyi	Kawaala	Komamboga	Kisugu	Naguru	Kiswa	Murchison Bay	Kitebi	Mengo	Nsambya
Location *	Urban	Urban	Rural	Urban	Urban	Urban	Peri-urban	Peri- urban	Urban	Urban
Type	Public	Public	Public	Public	Public	Public	Public	Public	Private	Private
No. of health workers/volunteers at TB clinic	2/5	2/3	2/2	2/2	2/2	2/2	2/2	2/2	2/2	2/2
No. of health centre beds	32	16	16	16	250	16	100	16	250	250
Average no. of daily patient visits at TB clinic	150	40	20	30	50	40	20	20	40	30
DM services before the study	None	None	None	None	Yes	None	None	None	Yes	Yes

*Urban = central business area; Peri-urban = slightly away from central business area; Rural = furthest away from central business area. TB, tuberculosis; DM, diabetes mellitus

**Table 2.** Diabetes mellitus screening among TB patients in Kampala, Uganda, from 2016–2017 (10 centres combined).

Registered TB patients screened for and diagnosed with DM	Number
Number of registered TB patients	4,590
Number with known DM	26
Number to be screened with RBG	4,564
Number (%) screened with RBG	4,016 (88.0%)
Number with RBG ≥ 6.1 mmol/l and to be screened with FBG	1,312
Number (%) screened with FBG	1,093 (83.3%)
Number with FBG ≥ 7.0 mmol /l	66
Number (%) of newly diagnosed with DM *	66 (71.7%)
Number (%) with known and newly diagnosed DM	92 (2.3%)

* Percentage of newly diagnosed DM among total number of DM

TB, tuberculosis; DM, diabetes mellitus; RBG, random blood glucose; FBG, fasting blood glucose

The number of registered TB patients in reporting periods one, two, three and four were 372, 1,576, 1,364 and 1,278, respectively. The proportion of patients screened with RBG (number of screened with RBG/number of registered TB patients) were 83.1%, 83.8%, 91.6% and 89.0%, respectively, over the four reporting periods, but the absolute number of TB patients screened with RBG in the first reporting period was 7.7%. Of the number of TB patients with RBG ≥6.1mmol/l, the proportion of screened with FBG (number of screened with FBG/number of RBG ≥6.1mmol/l) were 81.4%, 89.8%, 95.7% and 63.9% over the four reporting periods.

The proportion of patients screened with RBG and FBG varied from 78.9% to 94.5%, and from 58.2% to 100.0%, respectively, among the health centres ( Table 3). The proportion of patients screened with RBG was significantly higher in the health centres located in peri-urban areas compared with the centres in urban areas (OR 1.50, 95% CI 1.12-1.99, *P*=0.006); and in those without DM services compared with those with DM services (OR 1.35, 95% CI 1.11-1.63, *P*=0.002) ( Table 4). Of the patients with RBG ≥6.1mmol/l, the proportion screened with FBG was significantly higher in private health centres (OR 2.16, 95% CI 1.26-3.69, *P*=0.005) and those without DM services (OR 1.96, 95% CI 1.44-2.67, *P*<0.001) than that in public health centres and in those with DM services.

**Table 3.** Screening TB patients for DM at the 10 health centres: data combined for the four reporting periods.

	Kisenyi	Kawaala	Komamboga	Kisugu	Naguru	Kiswa	Murchison Bay	Kitebi	Mengo	Nsambya
No. of TB patients registered over the four reporting periods	1,183	593	329	339	555	299	319	324	313	336
No. with known diagnosis of DM	9	3	1	2	3	0	1	0	3	4
No. needing to be screened with RBG	1,174	590	328	337	552	299	318	324	310	332
No. (%) screened with RBG	1,107 (94.3)	466 (79.0)	280 (85.4)	298 (88.4)	468 (84.8)	257 (86.0)	293 (92.1)	292 (90.1)	293 (94.5)	262 (78.9)
No. with RBG ≥ 6.1 mmol/l	346	211	99	94	153	72	76	87	77	98
No. (%) screened with FBG	281 (81.2)	170 (80.6)	91 (91.9)	94 (100.0)	89 (58.2)	70 (97.2)	66 (86.8)	73 (83.9)	72 (93.5)	87 (88.8)
No. with FBG ≥ 7.0 mmol/l referred to DM services	19	5	9	1	6	3	3	3	6	11
No. newly diagnosed with DM *	19	5	9	1	6	3	3	3	6	11
No. (%) with known or newly diagnosed DM	28 (2.5)	8 (1.7)	10 (3.6)	3 (1.0)	9 (1.9)	3 (1.2)	4 (1.4)	3 (1.0)	9 (3.1)	15 (5.7)

* Percentage of newly diagnosed DM among total number of DM

TB = tuberculosis; DM = diabetes mellitus; RBG = random blood glucose; FBG = fasting blood glucose

**Table 4.** Screening TB patients for DM at the 10 health centres: data combined for the four reporting periods stratified by type, location and available of DM service.

Characteristics of the health centres	No. of TB patients registered	No. (%) screened with RBG	No. with RBG≥ 6.1 mmol/l	No. (%) screened with FBG	No. (%) with known or newly diagnosed DM	% of newly diagnosed DM *
**Type**						
Public (Reference)	3,941	3,641 (87.8)	1,137	934 (82.2)	68 (2.0)	72.1
Private	649	555 (85.5)	175	159 (90.9)	24 (4.3)	70.8
OR (95% CI)		0.82 (0.65-1.04)		2.16 (1.26-3.69)	2.26 (1.40-3.62)	0.94 (0.34-2.63)
*P*-value		0.101		0.005	0.001	0.909
**Location**						
Urban (Reference)	3,618	3,151 (87.1)	1,050	863 (82.2)	75 (2.4)	68.0
Peri-urban	643	585 (91.0)	163	139 (85.5)	7 (1.2)	85.7
OR (95% CI)		1.50 (1.12-1.99)		1.26 (0.79-1.99)	0.50 (0.23-1.08)	2.82 (0.32-24.77)
*P*-value		0.006		0.334	0.079	0.349
Rural	329	280 (85.4)	99	91 (91.9)	10 (3.6)	90.0
OR (95% CI)		0.85 (0.62-1.17)		2.47 (1.18-5.17)	1.52 (0.78-2.97)	4.24 (0.51-35.36)
*P*-value		0.307		0.017	0.222	0.182
**Available DM** **service**						
Yes (Reference)	1,204	1,023 (85.0)	328	248 (75.6)	33 (3.2)	69.7
None	3,386	2,993 (88.4)	984	845 (85.9)	59 (2.0)	72.9
OR (95% CI)		1.35 (1.11-1.63)		1.96 (1.44-2.67)	0.60 (0.39-0.93)	1.17 (0.46-2.99)
*P*-value		0.002		<0.001	0.022	0.745

* Percentage of newly diagnosed DM among total number of DM

TB = tuberculosis; DM = diabetes mellitus; RBG = random blood glucose; FBG = fasting blood glucose

A total of 92 TB patients were diagnosed with DM, accounting for a prevalence of 2.3%. The prevalence of DM in TB patients was higher in the private health centres (OR 2.26, 95% CI 1.40-3.62, *P*=0.001) and in those with DM services (OR 0.60, 95% CI 0.39-0.93, *P*=0.022). 

## Discussion

This is the first study to report on screening TB patients for DM in a primary health care setting in Uganda. We included both public and private facilities in urban, peri-urban and rural parts of Kampala to assess the feasibility of integrating DM screening into routine TB service. 

The overall prevalence of DM in the screened TB patients was 2.3% (92/4,016), which was higher than that in general population and our finding mirrored the findings of previous studies ^6, 17, 18, 20^, although it was lower than that reported by a study among hospitalized TB patients at a large referral hospital in an urban area of Uganda ^21^. However, using only FBG to diagnose DM can underestimate the prevalence of DM by as much as 50% when compared with the gold standard test, the oral glucose tolerance test, which is more accurate but unavailable at primary health care level ^22^. In addition, some of the facilities raised the FBG screening threshold to 7.0 mmol/l during the first 6–8 months due to misinterpretation of the screening algorithm. We therefore believe that the actual prevalence of DM among TB patients in Uganda might be higher than we have observed in this study. Of the 92 DM patients detected, 66 (71.7%) had not been diagnosed before this study. This strongly demonstrated the need for integrating DM screening into routine TB service ^5, 23^.

In the first six months, a small number of TB patients were screened with RBG, due to the long engagement of stakeholders followed by the cascade training. One health centre (Nsambya) did not send staff to attend the training and did not screen any patients in the first reporting period due to staff turn-over, a situation that should have been identified by training organizers, and this challenge required KCCA attention. The number of screened TB patients increased rapidly over the rest of the reporting periods, suggesting that the procedure was gradually accepted by staff, and we echo a similar finding in China ^17^. The proportion of patients screened with FBG declined over the last six-month period due to gluco-strips stock-out at the government medical stores. It was reassuring that the health centre staff largely followed the screening algorithm, although there were certain differences. Kisugu Health Centre ensured FBG screening for everyone with RBG ≥ 6.1mmol/l due to strong commitment of the staff. On the contrary, Naguru Health Centre managed to screen only 58.2% of the patients who needed an FBG test even though they had the same number of health workers, although they did have a slightly higher number of TB patient visits. It is necessary to understand reasons for missed opportunities in the DM screening cascade.

Private health centres screened higher proportions of patients with FBG and, subsequently, identified more DM patients than public facilities. Possible reasons for this could be that patients with a higher socio-economic status sought care in private health centres, which have well-motivated staff thanks to better remuneration. Similarly, staff in peri-urban and rural facilities screened higher proportions of patients with RBG and FBG than in urban areas. Murchison Bay is a health centre in peri-urban area operated by the Ministry of Internal Affairs with prisoners being the main source of patients. Given that most patients were confined in prison, they were available for RBG and FBG. In urban health centres there is more frequently congestion and longer waiting times than in rural centres. This may have caused some patients to miss their FBG tests. 

An intriguing finding that also requires a further study was that health centres with available DM services at time of the study, a feature that tends to reflect better health services, did not screen more patients with RBG and FBG compared with those without DM services. This was unexpected and not in line with the findings of other studies ^16^. Low screening at Naguru Health Centre could have contributed to this finding.

There were some service-related barriers identified in the project implementation: 1) lack of a sustainable supply of glucose testing strips, which caused insufficient screening for RBG and FBG; 2) lack of community support mechanisms to help trace patients who failed to return for FBG testing; 3) insufficient number of staff in the health centres caused by staff turn-over; 4) routine DM service was not available in some health centres.

This study had several limitations. Diagnosis of DM relied on FBG alone and we were unable to include oral glucose-tolerance tests, a more accurate test, as part of the screening cascade. This may have resulted in an underestimated prevalence of DM in TB patients ^21^. Blood samples for RBG and FBG were taken from capillary blood and measured with glucometers without quality assurance, rather than using venous blood specimens and tested with biochemical analyser. This may have caused a testing bias ^24^. RBG or FBG were measured only once immediately after TB registration, without any confirmatory test at the end of anti-TB treatment, and glycosylated haemoglobin (HbA1c) tests were not carried out, which is influenced less by infection ^25^. Therefore, we do not know whether any TB patient may have been diagnosed as having DM due to infection-induced hyperglycaemia. However, a previous study suggested that taking one blood sample for FBG immediately after TB diagnosis would be appropriate for majority of TB patients ^14^. We did not collect individual patient data, including socio-demographic data, and perform multivariate analysis and were unable to identify and control potential confounding factors. Finally, the selected health centers were only located in Kampala due to financial limitations and transportation barriers. This might have limited the representative value.

The strength of this study is that we implemented DM screening among a large number of TB patients in routine health service using mainly existing resources. Only training, meetings, support supervision, mentorship and provision of glucometers were supported. There were no staff or patient incentives. This is a key point for sustainability and scale up of integration of DM and TB services. Some challenges identified in the study need government attention and this indicates the need for collaboration between TB and non-communicable disease programs.

## Conclusion

This study revealed that prevalence of DM in TB patients is higher than that in the general population in Uganda. Screening TB patients for DM in a primary health care setting in Kampala with high HIV-associated TB epidemic appeared feasible. RBG and FBG are practical tools to be used for screening in routine program settings even though they may underestimate the actual situation. This study identified certain service-related factors that may be barriers and need to be addressed by relevant authorities. Inclusion of bi-directional screening in both TB and DM clinics could be considered. Every year, 44,000–47,000 persons with TB are registered in Uganda and if all were screened for DM, at least 1,748 additional persons with DM could be detected and enrolled to DM care. As early diagnosis and management for the two diseases will improve treatment outcomes and well-being of people, this is an important public health intervention.

## Data availability

### Underlying data

Figshare: Screening tuberculosis patients for diabetes mellitus in a routine program setting in Kampala, Uganda: a cross-sectional study. https://doi.org/10.6084/m9.figshare.8248625.v1 ^19^

This project contains the following underlying data:

- TB_DM DATA _Uganda 2016_2017.xlsx (raw data collected from health centres over the four reporting periods)
- Screening tuberculosis patients for diabetes mellitus in a routine program setting in Kampala, Uganda a cross-sectional study.docx (table of data processes)

Data are available under the terms of the Creative Commons Zero "No rights reserved" data waiver (CC0 1.0 Public domain dedication).
