# Supplementary material for: Screening tuberculosis patients for diabetes mellitus in a routine program setting in Kampala, Uganda: a cross-sectional study
Source: F1000Res. 2019 Oct 10;8:872. Originally published 2019 Jun 17. [Version 2] doi: 10.12688/f1000research.19279.2 (PMC6816448; doi:10.12688/f1000research.19279.2)
Supplement: Peer review history

# Peer review: Reviewer response for version 2

**DOI:** 10.5256/f1000research.22880.r54934

**Published:** 2019-10-24

**Authors:** Razia Kaniz Fatima

**Affiliations:** 1 National TB Control Program, Islamabad, Pakistan

I am satisfied with the revisions.

I confirm that I have read this submission and believe that I have an appropriate level of expertise to confirm that it is of an acceptable scientific standard.

# Peer review: Reviewer response for version 2

**DOI:** 10.5256/f1000research.22880.r54935

**Published:** 2019-10-21

**Authors:** Daniel C. Oshi

**Affiliations:** 1 Department of Community Health and Psychiatry, The University of West Indies, Mona, Kingston, Jamaica

The authors have addressed the concerns I raised in my first review. I am satisfied with the manner and extent of the revision.

I confirm that I have read this submission and believe that I have an appropriate level of expertise to confirm that it is of an acceptable scientific standard.

# Peer review: Reviewer response for version 1

**DOI:** 10.5256/f1000research.21133.r53770

**Published:** 2019-09-17

**Authors:** Daniel C. Oshi

**Affiliations:** 1 Department of Community Health and Psychiatry, The University of West Indies, Mona, Kingston, Jamaica

General Comment:

 The manuscript is on a very important public health issue, which is at the intersection between communicable and non-communicable diseases control. It is especially relevant because of the contextual issues of high TB and HIV burden in Uganda, the increasing burden of DM as a result of changing lifestyles and nutritional patterns, and limited resources in the country.

 Abstract: The abstract is well written and captures the essence of the paper.

 Introduction: The Introduction section is well written, supported with key epidemiological facts and figures on TB and DM, globally, in the Sub-Saharan African region and in Uganda. The authors concisely made a strong case for the rationale of the study, from the epidemiological and clinical perspectives and aptly noted the absence of policy on routine screening for TB and DM in health care settings as well as the paucity of evidence to guide the formulation of such a policy. Having created gaps in knowledge and policy that their paper will contribute toward addressing, the authors stated the objectives of their study.

 Methods: This section is, on the whole, well written, although it would have been useful to see a justification for the choice of only Kampala Capital City, especially because the research aimed to produce evidence for policy formulation for programmatic screening for TB and DM in the whole country, not just in Kampala Capital City.

 Results: This section is also well written. However, there are two issues of concern here. First, since this study was not just an academic exercise but a public health project, the authors should state measures they took, if any, to ensure that patients newly diagnosed with DM and referred to DM clinics actually reached and accessed care at DM clinics. Initial default is a major challenge in such referrals because some of the referred patients fail to go for the needed care. Second, it would have been useful to show the socio-demographic and clinical characteristics of all the patients versus those diagnosed with DM. This would have made this paper (the analysis, results and discussions) much stronger.

 Discussion: This is well written and acknowledged the limitation pointed out above about the non-collection and analysis of patient-level data.

 Conclusion: One key conclusion of the study is “Screening TB patients for DM in a primary health care setting in a resource-limited country with high HIV-associated TB epidemic appeared feasible.” This conclusion is apparently not supported by the geographical coverage of the study. The study was conducted only in Kampala City Capital. The inclusion of just one rural health centre, which is still part of the KCC is insufficient to be used to drawn conclusions regarding rural setting. The geographic spread and the socio-economic characteristics of the locations of almost all the health centres in the study therefore suggest that the conclusion should be drawn to Kampala City Council rather than to the entire country.

 Finally, the authors are commended for the effort in implementing this important research project.

I confirm that I have read this submission and believe that I have an appropriate level of expertise to confirm that it is of an acceptable scientific standard, however I have significant reservations, as outlined above.

# Response

**Published:** 2019-09-29

**Authors:** Yan Lin

**Affiliations:** International Union Against Tuberculosis and Lung Disease, China

Reviewer 2:

General Comment:

The manuscript is on a very important public health issue, which is at the intersection between communicable and non-communicable diseases control. It is especially relevant because of the contextual issues of high TB and HIV burden in Uganda, the increasing burden of DM as a result of changing lifestyles and nutritional patterns, and limited resources in the country.

**Response: Thank you.**

Abstract: The abstract is well written and captures the essence of the paper.

**Response: Thank you.**

Introduction: The Introduction section is well written, supported with key epidemiological facts and figures on TB and DM, globally, in the Sub-Saharan African region and in Uganda. The authors concisely made a strong case for the rationale of the study, from the epidemiological and clinical perspectives and aptly noted the absence of policy on routine screening for TB and DM in health care settings as well as the paucity of evidence to guide the formulation of such a policy. Having created gaps in knowledge and policy that their paper will contribute toward addressing, the authors stated the objectives of their study.

**Response: Many thanks.**

Methods: This section is, on the whole, well written, although it would have been useful to see a justification for the choice of only Kampala Capital City, especially because the research aimed to produce evidence for policy formulation for programmatic screening for TB and DM in the whole country, not just in Kampala Capital City.

**Response: Point well taken! We have restructured some relevant parts in the method as highlighted in yellow colour to give justification on selection of the health centers. In addition, we have added a sentence in the limitation as “Finally, the selected HCs were only located in Kampala due to financial limitations and transportation barriers. This might have limited the representative value.”**

Results: This section is also well written. However, there are two issues of concern here. First, since this study was not just an academic exercise but a public health project, the authors should state measures they took, if any, to ensure that patients newly diagnosed with DM and referred to DM clinics actually reached and accessed care at DM clinics. Initial default is a major challenge in such referrals because some of the referred patients fail to go for the needed care.

**Response: This is excellent advice, thank you. We have rephrased the last sentences in the first paragraph of the Results as “Ninety-two TB patients were diagnosed with DM, and of them, 66 (71.7%) were newly diagnosed DM and were referred to DM services. All DM patients arrived at the  DM clinics and received DM care except four patients who died shortly after TB registration.**

Second, it would have been useful to show the socio-demographic and clinical characteristics of all the patients versus those diagnosed with DM. This would have made this paper (the analysis, results and discussions) much stronger.

**Response: Thank you for the valuable advice. We agree.** **To well accept this point, we have rephrased the sentence in the limitation as “We did not collect individual patient data, including socio-demographic data, and perform multivariate analysis and were unable to identify and control potential confounding factors.”**

Discussion: This is well written and acknowledged the limitation pointed out above about the non-collection and analysis of patient-level data.

**Response: Thanks.**

Conclusion: One key conclusion of the study is “Screening TB patients for DM in a primary health care setting in a resource-limited country with high HIV-associated TB epidemic appeared feasible.” This conclusion is apparently not supported by the geographical coverage of the study. The study was conducted only in Kampala City Capital. The inclusion of just one rural health centre, which is still part of the KCC is insufficient to be used to drawn conclusions regarding rural setting. The geographic spread and the socio-economic characteristics of the locations of almost all the health centres in the study therefore suggest that the conclusion should be drawn to Kampala City Council rather than to the entire country.

Finally, the authors are commended for the effort in implementing this important research project.

**Response: Thank you for pointing this out. We have revised the sentence in the conclusion as “Screening TB patients for DM in a primary health care setting in Kampala with high HIV-associated TB epidemic appeared feasible.”**

# Peer review: Reviewer response for version 1

**DOI:** 10.5256/f1000research.21133.r49993

**Published:** 2019-06-19

**Authors:** Razia Kaniz Fatima

**Affiliations:** 1 National TB Control Program, Islamabad, Pakistan

The article is well written and addresses the Global issue of DM screening among TB Patients which is one the common co morbidity recognized with TB  that may affect the TB outcome.

 The points which need to be explained are:

1. The selection criteria of the centres and how public vs private centres were selected moreover was the urban rural representation was also considered or it was random?
  Moreover it was stated in abstract that health centres without DM screened larger number of patients while in methods it was stated the DM services were mainly in tertiary care hospitals which might create assumptions of large screening in tertiary care hospitals contrary to what was stated in abstract. Table 1 also shows the DM services to be already present in 3 out of 7 centres so this need to be better explained. The average number of daily patients screened was also less in these 3 centres so it might mean that tertiary care where already DM services are present were not part of study?
2. In discussion it was mentioned using only FBG to diagnose DM can underestimate the prevalence of DM by as much as 50% when compared with the gold standard test, the oral glucose tolerance test, which is more accurate but unavailable at primary health care level, so how this should spell out in the policy implication? When the program should plan to do this DM screening of TB patients, more focus should be on the availability of gold standard for FBG using Glucometer, this should also be explained.

I confirm that I have read this submission and believe that I have an appropriate level of expertise to confirm that it is of an acceptable scientific standard, however I have significant reservations, as outlined above.

# Response

**Published:** 2019-09-29

**Authors:** Yan Lin

**Affiliations:** International Union Against Tuberculosis and Lung Disease, China

Reviewer 1

The article is well written and addresses the Global issue of DM screening among TB Patients which is one the common co morbidity recognized with TB  that may affect the TB outcome.

The points which need to be explained are:

- The selection criteria of the centres and how public vs private centres were selected moreover was the urban rural representation was also considered or it was random?

**Response: Thank you for this valuable advice. As described in the site and setting, selection of the health centers was based on their location in the five administrative divisions of KCCA and representative of the health delivery system in Kampala as well as willingness of the staff to participate this study. It was however not random selected. We have rephrased the sentence as “These were high volume health facilities selected from different communities based on their ability to manage sufficient numbers of TB patients registered in the previous year and a daily TB patient visits** **≥** **20/day, availability of diagnostic and treatment facilities and willingness of the staff to participate in this study.”**

- Moreover it was stated in abstract that health centres without DM screened larger number of patients while in methods it was stated the DM services were mainly in tertiary care hospitals which might create assumptions of large screening in tertiary care hospitals contrary to what was stated in abstract. Table 1 also shows the DM services to be already present in 3 out of 7 centres so this need to be better explained. The average number of daily patients screened was also less in these 3 centres so it might mean that tertiary care where already DM services are present were not part of study?

**Response: Thank you for the question. The ten health centers recruited in this study are primary health care institutions. There was no any tertiary hospital involved in this study. Of the ten health centers, the health center without DM service screened large number of patients (both RBG and FBG) than those with DM service. **

- In discussion it was mentioned using only FBG to diagnose DM can underestimate the prevalence of DM by as much as 50% when compared with the gold standard test, the oral glucose tolerance test, which is more accurate but unavailable at primary health care level, so how this should spell out in the policy implication? When the program should plan to do this DM screening of TB patients, more focus should be on the availability of gold standard for FBG using Glucometer, this should also be explained.

**Response: Excellent advice! Thank you. We have added a sentences in the Conclusions as “RBG and FBG are practical tools to be used for screening in routine program settings even though they may underestimate the actual situation".**
